# Developmental Up-Regulation of Vesicular Glutamate Transporter-1 Promotes Neocortical Presynaptic Terminal Development

**DOI:** 10.1371/journal.pone.0050911

**Published:** 2012-11-30

**Authors:** Corbett T. Berry, Michael P. Sceniak, Louie Zhou, Shasta L. Sabo

**Affiliations:** Departments of Pharmacology and Neurosciences, Case Western Reserve University School of Medicine, Cleveland, Ohio, United States of America; CSIC-Univ Miguel Hernandez, Spain

## Abstract

Presynaptic terminal formation is a complex process that requires assembly of proteins responsible for synaptic transmission at sites of axo-dendritic contact. Accumulation of presynaptic proteins at developing terminals is facilitated by glutamate receptor activation. Glutamate is loaded into synaptic vesicles for release via the vesicular glutamate transporters VGLUT1 and VGLUT2. During postnatal development there is a switch from predominantly VGLUT2 expression to high VGLUT1 and low VGLUT2, raising the question of whether the developmental increase in VGLUT1 is important for presynaptic development. Here, we addressed this question using confocal microscopy and quantitative immunocytochemistry in primary cultures of rat neocortical neurons. First, in order to understand the extent to which the developmental switch from VGLUT2 to VGLUT1 occurs through an increase in VGLUT1 at individual presynaptic terminals or through addition of VGLUT1-positive presynaptic terminals, we examined the spatio-temporal dynamics of VGLUT1 and VGLUT2 expression. Between 5 and 12 days in culture, the percentage of presynaptic terminals that expressed VGLUT1 increased during synapse formation, as did expression of VGLUT1 at individual terminals. A subset of VGLUT1-positive terminals also expressed VGLUT2, which decreased at these terminals. At individual terminals, the increase in VGLUT1 correlated with greater accumulation of other synaptic vesicle proteins, such as synapsin and synaptophysin. When the developmental increase in VGLUT1 was prevented using VGLUT1-shRNA, the density of presynaptic terminals and accumulation of synapsin and synaptophysin at terminals were decreased. Since VGLUT1 knock-down was limited to a small number of neurons, the observed effects were cell-autonomous and independent of changes in overall network activity. These results demonstrate that up-regulation of VGLUT1 is important for development of presynaptic terminals in the cortex.

## Introduction

The cortical maps responsible for perception and behavior are comprised of a complex network of synapses. Understanding how these synapses form has been a major focus in developmental neurobiology, and abnormal synapse development may be linked to a number of neurological disorders, including autism, epilepsy, amblyopia, depression and schizophrenia [1]. Development of presynaptic terminals requires the coordinated delivery of the ensemble of synaptic vesicles (SV) and active zone (AZ) proteins that allow synaptic transmission to ensue [2], [3]. However, the mechanisms that regulate assembly of synaptic proteins at nascent presynaptic terminals remain unclear. Because activity is known to play a major role in the establishment and refinement of cortical circuits and sensory maps [4], [5], [6], delineating the role of neurotransmitters in presynaptic terminal assembly is of particular interest [7], [8], [9].

The predominant excitatory neurotransmitter in the cerebral cortex is glutamate. Glutamate is loaded into SVs via vesicular glutamate transporters, of which there are three known isoforms: VGLUT1, VGLUT2 and VGLUT3 [10]. Although they exhibit similar transport properties, each VGLUT isoform shows a unique spatio-temporal expression profile in the developing and adult brain. In the adult brain, VGLUT1 and VGLUT2 are expressed in complementary regions [11], [12], [13], [14]: VGLUT1 expression is high in the cerebral cortex, hippocampus, and cerebellar cortex, while VGLUT2 expression appears highest in neurons of the brainstem and deep cerebellar nuclei. In the developing brain, the two isoforms are co-expressed in individual hippocampal and cortical neurons [15], [16], [17]. Several studies have provided evidence that, in cortex and hippocampus, VGLUT1 and VGLUT2 can reside in the same presynaptic terminals [11], [15], [17], [18] and synaptic vesicles [19]. However, others have suggested that VGLUT1 and VGLUT2 are targeted to separate terminals, even when expressed in the same neuron [16].

Previous characterizations of the temporal expression of VGLUT1 and VGLUT2 in the cerebral cortex and hippocampal sections indicate a developmental switch from VGLUT2 to VGLUT1. VGLUT2 is expressed at birth and decreases during development, while VGLUT1 is absent at birth and increases steadily throughout postnatal development [11], [17], [20]. Therefore, the relative contribution of VGLUT1 to excitatory synaptic transmission increases significantly and that of VGLUT2 decreases during the first 3 weeks postnatal [16].

Several lines of evidence suggest that VGLUT1 expression may be important for presynaptic terminal development. First, even as early as P5– during early stages of cortical circuit development – VGLUT1 co-localizes with other synaptic vesicle proteins, such as synapsin and synaptophysin [20], [21]. In glutamatergic neurons, SVs and their precursors express vesicular glutamate transporters and are capable of releasing glutamate along axons and from axonal growth cones, even prior to synapse assembly [22], [23], [24]. In addition, VGLUT1 levels directly influence the amount of vesicular glutamate loading and release [25], [26], and it has recently been shown that activation of glutamate receptors regulates the accumulation of synaptic proteins at presynaptic terminals [27]. Furthermore, glutamate is a potent regulator of axonal filopodial motility, which is important for generation of contacts with dendrites [28], [29].

Here, we test the hypothesis that the developmental increase in VGLUT1 is important for presynaptic terminal development using confocal microscopy. We provide a quantitative analysis of the relative spatial and temporal expression dynamics of VGLUT1 and VGLUT2 at individual presynaptic terminals of primary visual cortical neurons during the first two weeks in culture. Our data provide evidence for a developmentally regulated increase in both the fraction of terminals expressing VGLUT1 and the levels of VGLUT1 at individual synapses. In contrast, VGLUT2 expression decreased during the same time period. Using shRNA against VGLUT1 to inhibit the developmental increase in VGLUT1 without altering overall network activity, we observed distinct changes in both the accumulation of synaptic vesicle proteins and the density of presynaptic terminals. These data support the hypothesis that VGLUT1 can cell-autonomously regulate the accumulation of synaptic proteins at nascent synapses.

## Materials and Methods

All studies were conducted with an approved protocol from the Case Western Reserve University Institutional Animal Care and Use Committee, in compliance with the National Institutes of Health guidelines for care and use of experimental animals.

### Neuronal Cultures and Transfection

Neurons were prepared by dissociation from visual cortices of 0–3 day old Long Evans or Sprague Dawly rats [22], [30] then plated (3–4 X 10^4^ cells/ml) on a confluent monolayer of cortical astrocyes. Neuron cultures were maintained in Neurobasal-A medium with glutamax and B27 supplement. Astrocyes were grown on coverslips coated with collagen and poly-L-lysine and maintained in Minimum Essential Medium containing glutamax, 10% fetal calf serum, glucose (0.6%), N2 and penicillin-streptomycin. At 2–4 days in vitro (DIV), cultures were treated with anti-mitotic (5′fluoro-2′-deoxy-uridine/uridine). Neurons were transfected at 5–6 DIV using calcium-phosphate [31], [32]. Cell culture reagents were from Invitrogen and other chemicals were from Sigma unless otherwise indicated.

### RNAi Constructs

Plasmids expressing shRNAs targeting VGLUT1 (rat, Accession # NM_053859) were designed using OligoEngine design services and constructed in pSUPER.neo+GFP, which co-expresses GFP (OligoEngine). Three sequences were evaluated for their ability to decrease VGLUT1 expression: (#1634) 5′-GATCCCCCAGCACAGTTCAGCCTCCATTCAAGAG ATGGAGGCTGAACTGTGCTGTTTTTA-3′, (#270) 5′-GATCCCCGTCAACAACAGTACAACCCTTCAAGAGAG GGTTGTACTGTTGTTGACTTTTTA-3′, and (#514) 5′-GATCCCCTCATCTTCGTGAGGATCCTTTCAAGAGAA GGATCCTCACGAAGATGATTTTTA-3′. Only #270 resulted in significant knockdown of VGLUT1 expression and was, therefore, used for the experiments described here. This sequence is specific for rat VGLUT1 to allow rescue with human VGLUT1. The negative control was pSUPER.neo+GFP containing a non-effective scrambled shRNA (SCR).

### Immunofluorescence and Confocal Imaging

Neuronal cultures were fixed (15 minutes, 25C) with 4% paraformaldehyde in 0.1 M phosphate-buffered saline containing 4% sucrose, permeabilized with 0.2% Triton X-100, and blocked with 10% horse serum or bovine serum albumin (BSA). Primary antibody labeling was in 3% horse serum or BSA overnight at 4C. Primary antibodies were: mouse anti-synaptophysin (Sigma), rabbit anti-synapsin (Chemicon, Synaptic Systems); rabbit anti-VGLUT2 (Synaptic Systems); guinea pig anti-VGLUT1, chicken anti-GFP (Chemicon); and mouse anti-PSD95 (NeuroMab). Secondary antibodies were Alexa-fluor conjugates (Invitrogen). Coverslips were mounted in fluoromount (Fisher) containing 1,4-diazabicyclo(2.2.2)octane as an anti-fade.

Imaging was performed on a Nikon C1 Plus confocal system on a Nikon Ti-E inverted microscope with 488Ar, 543HeNe and 633HeNe lasers and 40X 1.0NA and 0.95NA oil and air objectives, respectively. 3-D image stacks were collected using EasyC1 software (Nikon) with 2X Kalman averaging and a pixel size of 80–150 nm. Each channel was imaged separately to avoid bleed-through. On average, VGLUT1 appeared brighter than VGLUT2, consistent with previous observations [11]. Imaging parameters were optimized independently for each channel to maintain fluorescence within the linear range while maximizing intensity resolution. Parameters were then kept constant throughout each experiment.

### Analysis

Automated image analysis was performed using custom written macros in ImageJ. Maximum intensity Z-projections were made then background-subtracted using a rolling ball algorithm (an Image-J function). For VGLUT1 knockdown experiments, GFP images were used to select the axon. For other experiments, all puncta in the image were analyzed. Fluorescent puncta were automatically selected in the axon based on image statistics. In most cases, puncta were selected using synaptophysin or synapsin labels. In general, puncta were selected based on having a solid cluster of pixel intensities greater than 2 standard deviations above the mean intensity of the entire projection image. Puncta intensities and densities were measured in ImageJ, and measurements were transferred to Matlab (Mathworks, Natick, MA) for quantification using custom-written functions. All quantitative measurements were performed on 16-bit images. For analysis of colocalization, a given signal was deemed present at a punctum if the mean intensity for that punctum was above background. Background was defined as the mean intensity plus the mean standard deviation (as measured in ImageJ) for the 5 DIV images (whole images) for each experiment set. Histograms of all puncta intensities were used to verify these calculated cutoff values. For quantification of VGLUT1 fluorescence intensity in knockdown experiments, puncta were selected using synaptophysin or synapsin labels and VGLUT1 cutoff values for both control and knockdown puncta were derived from neurons transfected with control shRNA. In order to both combine data from multiple experiments and better illustrate the magnitudes of the observed changes, most data were normalized as follows: for time course experiments, data were normalized by dividing by the mean value for 5 DIV puncta. When values are given for a subset of puncta (e.g. only puncta expressing VGLUT1), normalization is to the mean of all 5 DIV puncta in order to maintain the observed differences between populations. For VGLUT1 knockdown experiments, data were normalized to the mean value of the control scrambled puncta. Data are presented as normalized mean +/− SEM. Statistical tests were performed in Matlab using the Statistics Toolbox. The following tests were used where appropriate, as indicated in the text: ANOVA with F-test, ANOVA with Tukey’s honestly significant difference (HSD) multiple comparison test, or Analysis of Covariance (ANOCOVA) with multiple comparisons.

## Results

### Colocalization and Relative Abundance of VGLUT1 and VGLUT2 in Individual Neocortical Presynaptic Terminals during Early Postnatal Development

**Figure 1:**
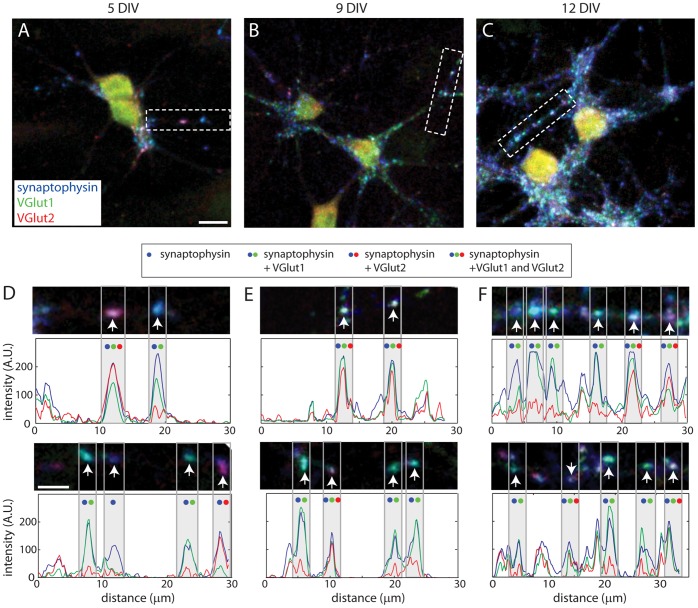
Throughout early postnatal development, cortico-cortical synapses are observed with VGLUT1, VGLUT2 or both VGLUT1 and VGLUT2. (**A, B and C**) Images of 5, 9 and 12 DIV neurons immunolabeled for synaptophysin (*blue*), VGLUT1 (*green*) and VGLUT2 (*red*). Scale bars, 10 µm. (**D, E and F**) Higher magnification images. The images of the top row in (D–F) are from the regions indicated by the dashed boxes in A-C. Scale bars, 5 µm. *Arrows* indicate the positions of presynaptic puncta in each image. Below each image, an intensity profile is shown, depicting the intensity of synaptophysin (*blue*), VGLUT1 (*green*), and VGLUT2 (*red*) along the axon in the image. The peaks that correspond to the presynaptic puncta, indicated by the *arrows* in the images, are highlighted with *gray bars*. *Blue, green and red dots* above each peak indicate whether synaptophysin (*blue*), VGLUT1 (*green*) or VGLUT2 (*red*) are found at each terminal, as indicated by the key.

The relative roles of VGLUT1 and VGLUT2 in excitatory synaptic transmission are determined by both the fraction of terminals that express VGLUT1 and VGLUT2 and their relative expression at individual terminals. It was previously shown that VGLUT1 expression is developmentally up-regulated while VGLUT2 is down-regulated [11], [17], [20]. To determine the extent to which these developmental changes occur through changes in the fraction of terminals expressing VGLUT1 and VGLUT2 or their expression levels at individual terminals of cortical neurons, we quantified the dynamics of the expression and co-localization of VGLUT1 and VGLUT2 at individual terminals during synapse formation. Neurons cultured from P0–P2 rat visual cortex were immunolabeled for VGLUT1, VGLUT2, and the integral SV protein synaptophysin at 5, 8–9 and 12DIV then imaged using multi-channel fluorescence confocal microscopy. Synaptophysin puncta were selected by an automated algorithm to identify presynaptic sites and will be referred to as presynaptic puncta below. As expected, the density of presynaptic puncta increased dramatically during this time (**Figures 1A–C** **and** **2A**).

**Figure 2:**
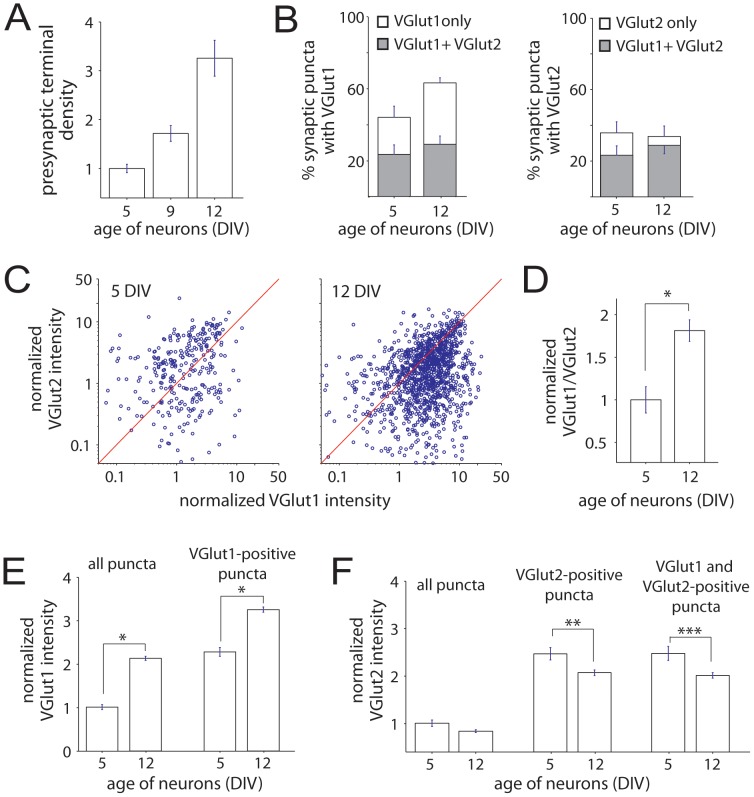
Expression of VGLUT1 increases while VGLUT2 decreases at individual presynaptic puncta during postnatal synapse development. (**A**) Synapse density increased dramatically between 5 and 12 DIV (normalized to 5DIV). (**B**) *Left,* As synapses developed, the percentage of presynaptic puncta that express VGLUT1 increased, as indicated by the *full bars*. The percentage of puncta that express VGLUT1 but not VGLUT2 also increased (*white portion of the bars*), while the percentage of puncta that expressed both VGLUT1 and VGLUT2 did not change (*grey portion of the bars*). *Right,* During the same time period, the percentage of presynaptic puncta that express VGLUT2 did not increase (*full bars)*, and the percentage of puncta that express VGLUT2 but not VGLUT1 was slightly, but not significantly, reduced (*white portion of the bars*). (**C**) Scatter plots of VGLUT1 versus VGLUT2 at 5 (*left*) and 12 (*right*) DIV. At all ages, puncta were observed that expressed only VGLUT1 (*not shown*), only VGLUT2 (*not shown*) or both VGLUT1 and VGLUT2 (*circles*). (**D**) The ratio of VGLUT1 to VGLUT2 at individual puncta increased during synapse development. Only puncta that expressed VGLUT1 and VGLUT2 were included in this analysis. (**E–F**) The mean intensity of VGLUT1 increased (E) while VGLUT2 decreased (F) at presynaptic puncta during early postnatal development. The increase in VGLUT1 was observed when all presynaptic puncta (E, *left*) or only VGLUT-1-positive puncta were analyzed (E, *right*). Similarly, the modest decrease in VGLUT2 was present regardless of whether all puncta (F, *left*), only VGLUT2-positive puncta (F, *middle*) or only VGLUT1- and VGLUT2-positive puncta (E, *right*) were analyzed. In (C–F), data were normalized to the mean value at 5 DIV and are presented as the mean +/− standard error. *, p<0.0001. **, p<0.01. ***, p<0.001. At 5DIV, n = 1176 total synaptophysin-positive puncta from 10 images in 3 experiments. At 12 DIV, n = 3649 total puncta from 15 images in 3 experiments.

At all ages studied, presynaptic puncta were present that expressed (i) only VGLUT1, (ii) only VGLUT2, or (iii) both VGLUT1 and VGLUT2 (**Figure 1**). In addition, presynaptic puncta were present that expressed neither: these may correspond to inhibitory terminals. The fraction of presynaptic puncta that expressed VGLUT1 increased steadily as synapse development proceeded. At 5 DIV, corresponding to the beginning of a period of intense excitatory synapse formation in the cortex, 43.7+/−6.2% of synaptophysin-labeled puncta (**Figure 2B**; n = 1176 synaptophysin puncta from 10 images) expressed detectable levels of VGLUT1. At 9 DIV, during the peak of excitatory synapse formation, 52.8+/−5.1% of synaptophysin puncta expressed VGLUT1 (n = 3205 synaptophysin puncta from 18 images). At 12 DIV, VGLUT1 was observed at 62.8+/−2.9% of synaptophysin puncta (**Figure 2B**; n = 3649 synaptophysin puncta from 15 images). In contrast, the percentage of presynaptic puncta that expressed VGLUT2 did not increase during synapse formation. At 5 and 9 DIV, 35.8+/−6.2% and 32.3+/−3.4% of synaptophysin-positive puncta were also VGLUT2 positive. Similarly, at 12 DIV, the percentage of synaptophysin puncta that expressed detectable VGLUT2 was 33.8+/−5.8% (**Figure 2B**).

Since puncta were observed that expressed both VGLUT1 and VGLUT2, the percentage of synaptophysin-positive puncta that expressed both VGLUT1 and VGLUT2 was also quantified. At 5 DIV, 23.2+/−5.3% of synaptophysin-positive puncta expressed both VGLUT1 and VGLUT2 (**Figure 2B**), while at 9 DIV, VGLUT1 and VGLUT2 were expressed at 24.6+/−3.0% of puncta. At 12 DIV, the percentage of presynaptic puncta that co-expressed VGLUT1 and VGLUT2 was 28.8+/−4.7% (**Figure 2B**), quantitatively similar to the levels of colocalization (∼25%) reported previously for mature cortical neurons [17]. In addition, 59.5+/−4.1% of VGLUT1 puncta (n = 462 puncta from 11 images) and 21.5+/−3.4% of VGLUT2 puncta (n = 920 puncta from 12 images) were associated with the postsynaptic marker, PSD95, already at 5 DIV, indicating that they were at synapses (data not shown). At 12 DIV, the percentage of VGLUT1 puncta associated with PSD95 was similar (59.2+/−4.5%; n = 3649 puncta from 12 images), while the percentage of VGLUT2 puncta at synapses increased to 30.8+/−4.2% (n = 2017 puncta from 12 images).

To determine whether the relative expression of VGLUT1 and VGLUT2 changes at individual terminals as synapses develop, the intensities of both were quantified in the same terminals. During synaptogenesis, the apparent ratio of VGLUT1 to VGLUT2 increased at puncta that expressed both VGLUTs (**Figure 2C**). Although the absolute intensities of VGLUT1 and VGLUT2 cannot be quantitatively compared when labeling with two different antibodies, changes in their relative intensities over time can be. Therefore, to quantitatively compare the relative expression of VGLUT1 and VGLUT2 over time, the ratio of VGLUT1 to VGLUT2 was determined for each presynaptic punctum then normalized to the mean ratio at 5 DIV. As neurons matured, the ratio of VGLUT1 to VGLUT2 increased significantly at individual terminals (**Figure 2D**; 81.3+/−12.8% increase between 5 and 12 DIV; p<0.0001, ANOVA with Tukey’s HSD test; n = 293 and 1223 puncta for 5 and 12 DIV, respectively).

This increase in the ratio of VGLUT1 to VGLUT2 at individual presynaptic terminals could be a result of increased VGLUT1, decreased VGLUT2 or both. To distinguish between these possibilities, we compared the developmental changes in VGLUT1 and VGLUT2 at individual terminals. When VGLUT1 was measured at all synaptophysin-positive puncta, the mean intensity of VGLUT1 increased during early stages of synapse development (**Figure 2E**). Between 5 and 12 DIV, the intensity of VGLUT1 at synaptophysin-positive puncta increased by 110.2+/−8.5% (p<0.0001; n = 1176 and 3649 puncta for 5 and 12 DIV, respectively). Because this apparent increase in VGLUT1 could be influenced by the increased percentage of presynaptic puncta that expressed VGLUT1, the intensity of VGLUT1 was then quantified only at terminals that were both synaptophysin- and VGLUT1-positive. At these presynaptic puncta, the intensity of VGLUT1 increased by 96.8+/−13.7% between 5 and 12 DIV (**Figure 2E**; p<0.0001; n = 511 and 2375 for 5 and 12 DIV, respectively). A similar increase in VGLUT1 intensity was observed when puncta were chosen using the VGLUT1 signal rather than synaptophysin. In addition, this increase in VGLUT1 expression was also apparent when analysis was limited to puncta that were associated with the excitatory postsynaptic marker PSD95, indicating that levels of VGLUT1 expression are increased at synapses and not just at very young presynaptic puncta (p = 0.0061; n = 264 and 1949 puncta from 11 and 12 images for 5 and 12 DIV, respectively).

In contrast, the intensity of VGLUT2 at all synaptophysin-positive presynaptic puncta decreased 16.8+/−6.7% during the same period (**Figure 2F**; p<0.01, ANOVA with Tukey’s HSD test). When VGLUT2 intensity was quantified at only puncta that were VGLUT2-positive, a decrease in VGLUT2 expression was also seen (**Figure 2F**; p<0.01 by ANOVA; n = 461 and 1413 for 5 and 12 DIV, respectively). The reduction in VGLUT2 expression was most prominent at puncta that expressed both VGLUT1 and VGLUT2 (**Figure 2F**; p = 0.001; n = 293 and 1223 for 5 and 12 DIV) and was also seen when analysis was restricted to puncta that contacted PSD95-positive postsynaptic densities. Because VGLUT1 increased while VGLUT2 decreased as synapses developed, we focused on VGLUT1 for the rest of the study.

### Relationship between the Expression of VGLUT1 and Other Synaptic Vesicle Proteins at Individual Presynaptic Terminals of Developing Neocortical Neurons

**Figure 3:**
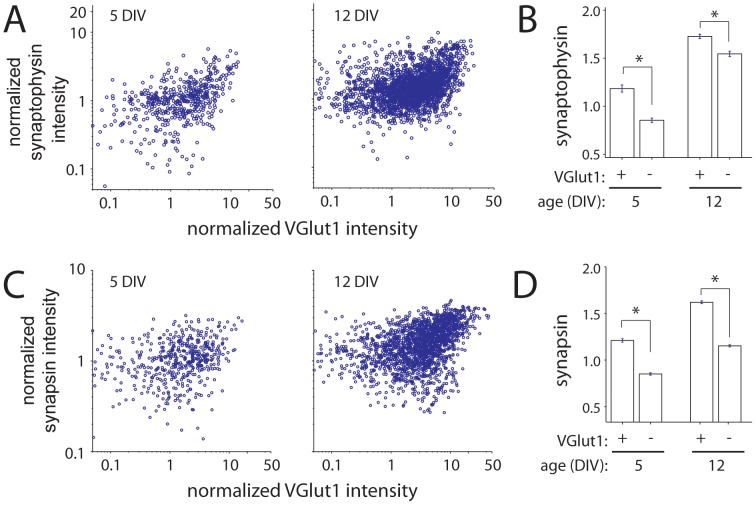
Correlation between age-dependent increases in presynaptic VGLUT1 and other synaptic vesicle proteins. (**A**) Scatter plots of VGLUT1 versus synaptophysin at 5 (*left*) and 12 (*right*) DIV. For VGLUT1-positive puncta, the Pearson’s correlation coefficient for the intensities of VGLUT1 and synaptophysin at presynaptic puncta was significant at 5 to 12 DIV (5 DIV: r = 0.568 with a 95% confidence interval of 0.506–0.624; 12 DIV: r = 0.418 with a 95% confidence interval of 0.385–0.451, p<0.0001 for both 5 and 12 DIV). At 5DIV, n = 511 synaptophysin and VGLUT1-positive puncta from 10 images in 3 experiments. At 12 DIV, n = 2375 puncta from 15 images in 3 experiments. (**B**) The mean intensity of synaptophysin was higher at puncta expressing VGLUT1 at both 5 and 12 DIV. Values were normalized to synaptophysin intensity of all presynaptic puncta at 5 DIV. (**C**) Scatter plots of VGLUT1 versus synapsin for synapsin and VGLUT1-positive presynaptic puncta (*circles*) at 5 (*left*) and 12 (*right*) DIV. The Pearson’s correlation coefficient for the intensity of VGLUT1 and synapsin at presynaptic puncta was significant at both 5 and 12 DIV, with r = 0.378 with a 95% confidence interval of 0.341–0.413 at 5 DIV and r = 0.497 with a confidence interval of 0.475–0.518 at 12 DIV; p<0.0001 for both 5 and 12 DIV. At 5DIV, n = 880 synapsin and VGLUT1-positive puncta from 11 images in 3 experiments. At 12 DIV, n = 2923 puncta from 12 images in 3 experiments. (**D**) Similar to synaptophysin, the intensity of synapsin was higher at puncta that expressed VGLUT1 than puncta without VGLUT1. Values are normalized to synapsin intensity for all presynaptic puncta at 5 DIV. In B and D, data are presented as the mean +/− standard error. *, p<0.0001.

VGLUT1 is recruited to nascent synapses together with most other integral synaptic vesicle proteins via synaptic vesicle precursor organelles [23], [33]. This raises the question of whether the developmental increase in VGLUT1 at individual terminals is accompanied by a concomitant increase in other synaptic vesicle proteins at the same terminals. To address this, we compared the expression of VGLUT1 and synaptophysin, an integral synaptic vesicle protein, in individual terminals during synapse development. Although absolute levels of both proteins were variable across terminals, expression of synaptophysin appeared to be positively correlated with expression of VGLUT1 at individual presynaptic puncta throughout synapse development (**Figure 3A**). To quantify this correlation, correlation coefficients for synaptophysin and VGLUT1 expression were determined for all puncta that expressed VGLUT1 (n = 511 and 2375 puncta for 5 and 12 DIV, respectively). At 5 DIV, the Pearson’s correlation coefficient was r = 0.568 with a 95% confidence interval of 0.506–0.624; while at 12 DIV, r = 0.418 with a 95% confidence interval of 0.385–0.451 (p<0.0001 for both ages). To further examine this correlation, linear regressions of the data were made at each age. Although the absolute slope of the correlation is not meaningful since synaptophysin and VGLUT1 are detected by different antibodies, the slopes can be compared across development. The slope of the regression decreased 28% between 5 and 12 DIV (significant by ANOCOVA, p<0.01). Finally, when synaptophysin puncta were sorted based on their expression of VGLUT1, VGLUT1-positive puncta expressed significantly more synaptophysin than VGLUT1-negative puncta (**Figure 3B**). This difference was present at both 5 and 12 DIV (p<0.0001 at all ages, ANOVA with Tukey’s HSD test; n = 511 and 2375 for VGLUT1-positive puncta at 5 and 12 DIV, respectively; n = 665 and 1274 for VGLUT1-negative puncta at 5 and 12 DIV).

In addition to integral synaptic vesicle proteins, developing presynaptic terminals must acquire the molecular scaffold that anchors synaptic vesicles at terminals. To determine whether accumulation of synaptic vesicle scaffolding proteins, such as synapsin, also correlates with the developmental increase in VGLUT1, synapsin and VGLUT1 expression levels were compared at individual terminals during synapse development (**Figure 3C**). At 5 DIV, 42% of synapsin puncta expressed VGLUT1 (n = 2123 puncta from 22 images). The percentage of synapsin puncta that expressed VGLUT1 rose to 61% at 12 DIV (n = 4776 puncta from 18 images). Similar to synaptophysin, synapsin expression was positively correlated with VGLUT1 expression (r = 0.378 with a 95% confidence interval of 0.341–0.413 at 5 DIV and r = 0.497 with a confidence interval of 0.475–0.518 at 12 DIV; p<0.0001 for both ages). Although the strength of the correlation between synapsin and VGLUT1 expression appeared to increase during synapse development, the slopes of linear regressions of the data at 5 and 12 DIV were not significantly different (ANOCOVA; n = 880 and 2923 puncta at 5 and 12 DIV, respectively). In addition, synapsin expression was increased at VGLUT1-positive puncta when compared to VGLUT1-negative puncta throughout synapse development (**Figure 3D**; p<0.0001 at both 5 and 12 DIV; n = 880 and 2993 VGLUT1-positive puncta and 1241 and 1843 VGLUT1-negative puncta at 5 and 12 DIV, respectively). Together, the above data support the idea that expression of VGLUT1 and other synaptic vesicle proteins are correlated at the level of individual terminals.

**Figure 4:**
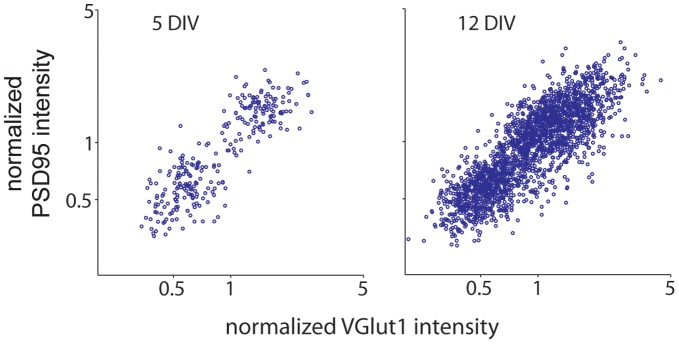
Presynaptic VGLUT1 and postsynaptic PSD95 expression levels are correlated at individual synapses at all ages studied. Scatter plots of VGLUT1 versus PSD95 at 5 (*left*) and 12 (*right*) DIV. The Pearson’s correlation coefficient for the intensities of VGLUT1 and PSD95 was significant at 5 to 12 DIV (5 DIV: r = 0.839 with a 95% confidence interval of 0.800–0.871; 12 DIV: r = 0.775 with a 95% confidence interval of 0.757–0.792, p<0.0001 for both 5 and 12 DIV). At 5DIV, n = 275 synaptophysin and VGLUT1-positive puncta from 11 images in 2 experiments. At 12 DIV, n = 2093 puncta from 12 images in 2 experiments.

Expression of presynaptic proteins and postsynaptic markers have been shown to be matched at individual synapses throughout postnatal development of hippocampal neurons [34]. Therefore, we determined whether the expression of VGLUT1 is also correlated with that of PSD95, an excitatory postsynaptic scaffolding protein, during postnatal development in cortical neurons. When expression levels of VGLUT1 and PSD95 were compared at individual synapses, the expression was well-correlated at both 5 and 12DIV (**Figure 4**; p<0.0001). The Pearson correlation coefficient was 0.839 at 5 DIV (with a 95% confidence interval of 0.800–0.871; n = 275 puncta from 11 images), while the correlation coefficient was 0.775 at 12 DIV (confidence interval = 0.757–0.792; n = 2093 puncta from 12 images). The slope of the linear regressions were slightly decreased at 12 DIV compared to 5 DIV (∼16%; p<0.01).

**Figure 5:**
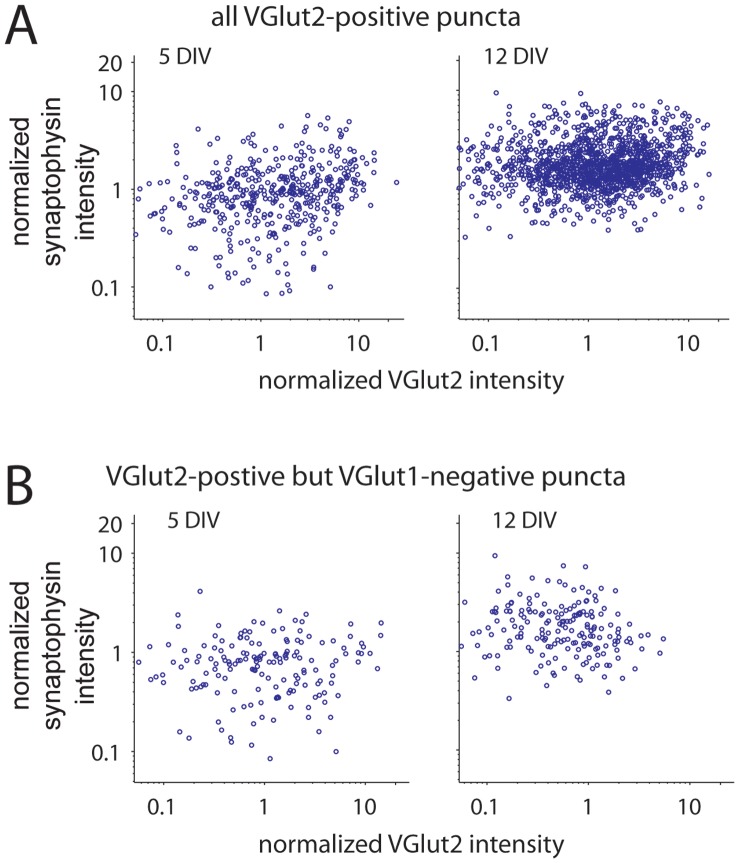
VGLUT2 does not correlate with other synaptic vesicle proteins. (**A**) Scatter plots of VGLUT2 versus synaptophysin at 5 (*left*) and 12 (*right*) DIV. For VGLUT2-positive puncta, the Pearson’s correlation coefficients were not significant at 5 or 12 DIV (5 DIV: r = 0.252 with a 95% confidence interval of 0.165−0.336, n = 461 puncta from 2 experiments; 12 DIV: r = 0.144 with a 95% confidence interval of 0.093−0.195, n = 1413 puncta from 2 experiments). (**B**) Scatter plots of VGLUT2 and synaptophysin at VGLUT2-positive but VGLUT1-negative terminals. VGLUT2 and synaptophysin were uncorrelated at both 5 and 12DIV (5DIV: r = 0.128, with a confidence interval of −0.024−0.274; 12DIV: r = −0.203, with a confidence interval of −0.335 to −0.062).

In contrast to the relationship observed between VGLUT1 and other synaptic proteins, expression of VGLUT2 did not appear to be correlated with expression of the synaptic vesicle protein synaptophysin (**Figure 5**). At 5, the correlation coefficient for VGLUT2 and synaptophysin was 0.252 (95% confidence interval of 0.165–0.336; n = 461 puncta). At 12 DIV, the correlation coefficient was 0.144 (95% confidence interval of 0.093–0.195; n = 1413 puncta). Because it seemed possible that VGLUT2 levels might correlate with other synaptic vesicle proteins preferentially at terminals that expressed VGLUT2 but not VGLUT1, this analysis was repeated with only VGLUT2-positive but VGLUT1-negative terminals. VGLUT2 and synaptophysin were uncorrelated at these terminals at both 5 and 12DIV (5DIV: r = 0.1281, with a confidence interval of −0.024−0.274; 12DIV: r = −0.203, with a confidence interval of −0.335 to −0.062). Therefore, the expression of synaptic proteins at individual synapses is correlated with expression of VGLUT1 but not VGLUT2. Based on this and the observation that VGLUT1 increased while VGLUT2 decreased as synapses developed, we focused on VGLUT1 for the rest of the study.

### Knock-down of VGLUT1 Expression during Postnatal Development Yields a Cell-autonomous Decrease in Expression of Synaptic Proteins at Individual Terminals

VGLUT1 expression levels influence the amount of glutamate released upon SV fusion [25], [35] and glutamatergic signaling regulates presynaptic protein recruitment [27]. Based on this and our observation that VGLUT1 expression is correlated with expression of other synaptic vesicle proteins at individual terminals, we hypothesized that the developmental increase in VGLUT1 is important for the accumulation of other synaptic vesicle proteins at terminals. To test our hypothesis, the developmental increase in VGLUT1 expression was prevented in a small fraction of neurons (approximately 1–10 neurons per coverslip) using shRNA-mediated knockdown of VGLUT1. By reducing VGLUT1 expression in only a small percentage of neurons, overall network activity was unperturbed and we could study the cell-autonomous effects of blocking the increase in VGLUT1 expression. Since VGLUT1 expression normally increases dramatically between 5 and 9 DIV (**Figure 2A**), neurons were transfected with VGLUT1 shRNA at 5 DIV then imaged at 8–9 DIV. Because neurons transfected with VGLUT1 shRNA co-express GFP, axons formed by knockdown neurons could be identified based on the GFP fill and terminals made by these axons could be selectively quantified.

**Figure 6:**
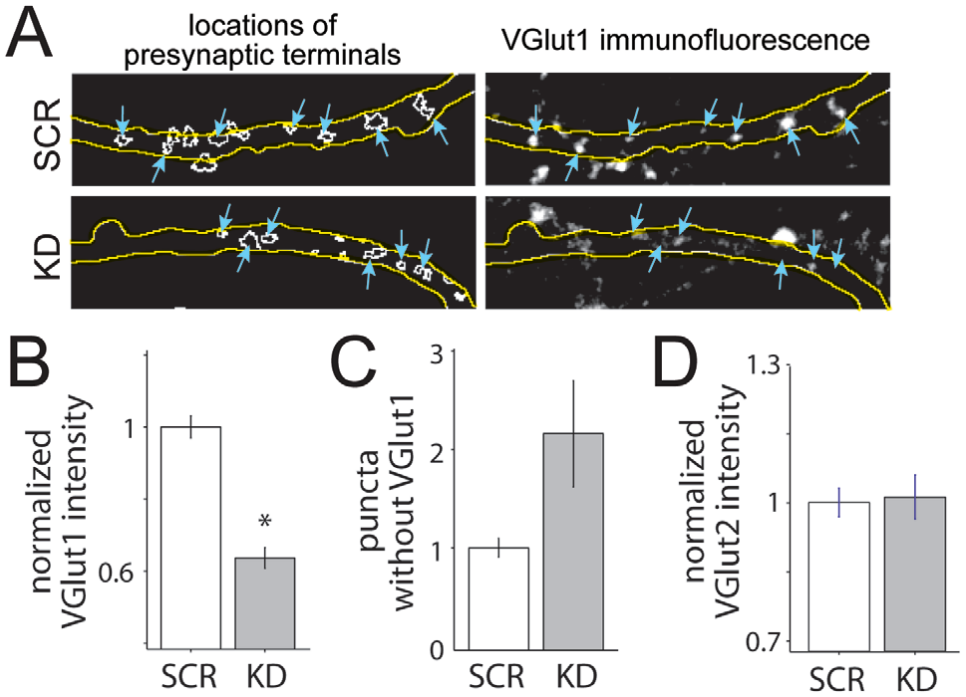
Transfection with VGLUT1 shRNA results in significant knockdown of VGLUT1 at presynaptic terminals. (**A**) Images of VGLUT1 presynaptic puncta near and within axons of cultured neurons transfected with control non-targeting shRNA (SCR, *top*) and VGLUT1-targeting shRNA (KD, *bottom).* Transfected neurons expressed GFP throughout the cell, and axons were identifiable by fluorescence and morphological characteristics (outlined in *yellow*). Synaptophysin-expressing presynaptic terminals were selected automatically via an imaging processing algorithm and identified as regions of interest (outlined in *white, left*). VGLUT1 immunofluorescence was measured within each region of interest. Presynaptic terminals within transfected axons are indicated with *blue arrows (left and right)*. (**B**) The mean intensity of VGLUT1 decreased significantly in KD puncta. *, p<0.001. (**C**) The percentage of puncta without VGLUT1 increased for the KD group (n = 1305 and 978 SCR and KD puncta, respectively, from 3 independent experiments; data are normalized to SCR). (**D**) VGLUT2 was not up-regulated in KD neurons (p = 0.8357, n = 440 and 246 puncta from 11 SCR and 14 KD axons, respectively).

To evaluate the level of VGLUT1 knockdown at presynaptic terminals, synaptophysin-positive presynaptic puncta were identified in GFP-expressing neurons, and VGLUT1 expression levels were quantified on a terminal-by-terminal basis. It was necessary to use a marker other than VGLUT1 to identify the terminals since it was expected that there would be an increase in VGLUT1-negative terminals. Three independent shRNAs were tested, and one of these (VGLUT1 shRNA #270) resulted in significant knockdown when compared to neurons transfected with a control scrambled shRNA (SCR). Therefore, VGLUT1 shRNA #270 was used for the remainder of the study, and neurons transfected with this shRNA will be referred to as KD neurons. Presynaptic puncta of KD neurons exhibited significantly decreased VGLUT1 expression (**Figure 6A–B**; 63.65+/−2.8% of control values; p<0.001), and substantially more puncta appeared to be VGLUT1-negative (**Figure 6A and C**; 2.1-fold more puncta without detectable VGLUT1), when compared to presynaptic puncta from control neurons (n = 1305 and 978 SCR and KD puncta). Importantly, VGLUT2 expression was not up-regulated to compensate for decreased VGLUT1 expression (**Figure 6D** **;** p = 0.8357; n = 440 SCR and 246 KD puncta), similar to previous observations in VGLUT1 knockout neurons **[15]**, **[16]**.

**Figure 7:**
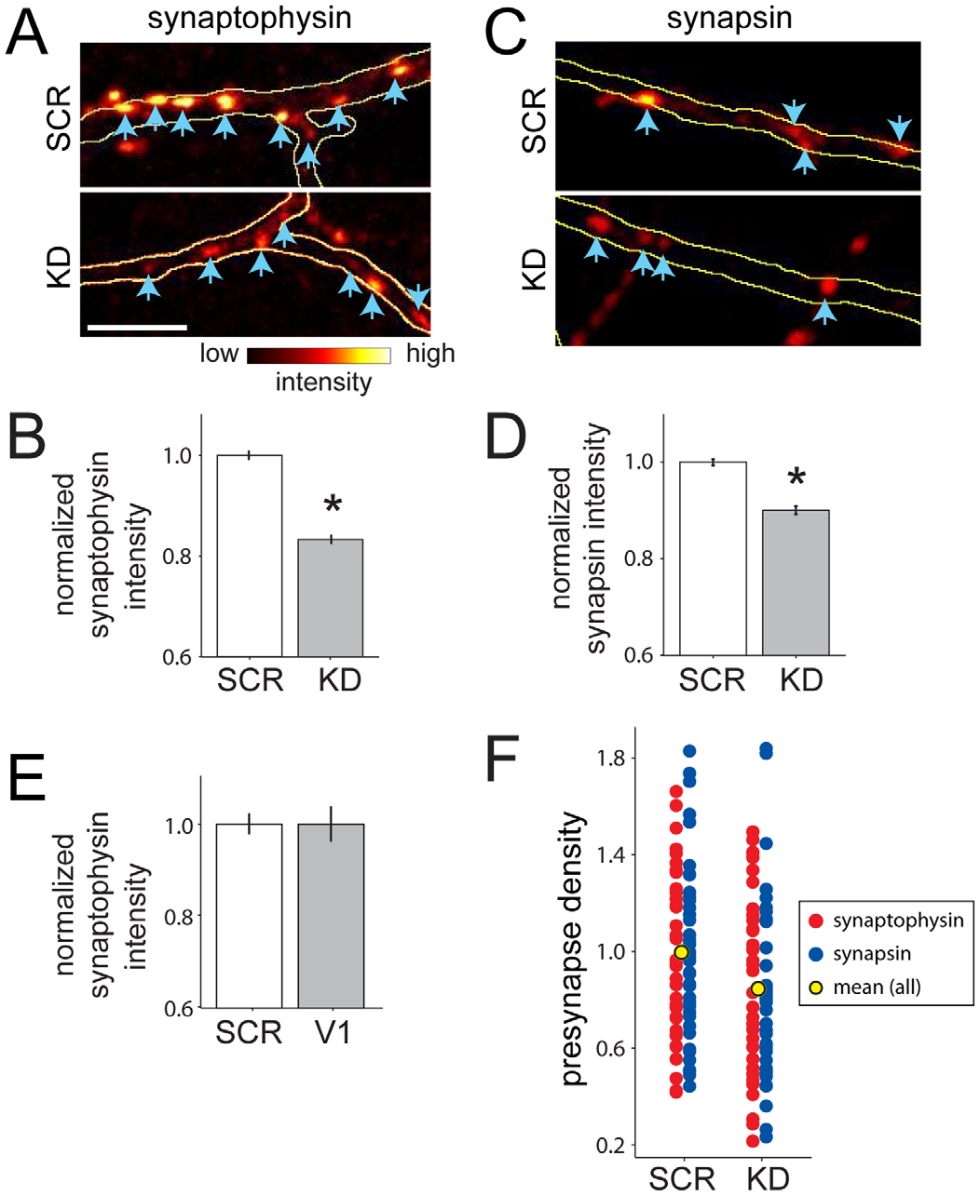
Knockdown of VGLUT1 results in a concomitant decrease in synaptic expression of synapsin and synaptophysin. (**A**) Images of fluorescently labeled synaptophysin puncta for control (SCR, *top)*, and VGLUT1 knockdown (KD, *bottom*) axons. Intensities were measured for the individual puncta (*blue arrows*) that fell within the confines of the fluorescently labeled axon (outlined in *yellow)*. (**B**), The mean synaptophysin intensity decreased significantly in KD neurons. *, p<0.0001 (n = 1305, and 978 puncta from 36 and 35 axons for SCR and KD, respectively, from 3 independent experiments). (**C**) Images of fluorescently labeled synapsin puncta for control (SCR, *top)*, and VGLUT1 knockdown (KD, *bottom*). The intensities of puncta (*blue arrows*) that fell within the confines of the fluorescently labeled axon (outlined in *yellow)* were quantified. (**D**) Mean synapsin intensity decreased significantly in KD neurons. *, p<0.0001 (n = 1802 and 1539 puncta from 57 and 51 axons for SCR and KD axons, respectively, from 3 experiments). (**E**) Synaptophysin was not decreased in KD neurons co-expressing VGLUT1 (*V1*, VGLUT1 expressing neurons; p = 0.940; n = 244 and 96 puncta from 13 and 9 axons for SCR and V1, respectively, from a representative experiment. The same results were observed in 3 independent experiments). (**F**) Plot of presynaptic density in SCR versus KD axons. Synaptophysin densities from individual images are indicated by *red dots* and synapsin densities are indicated by *blue dots.* The mean synapse densities for pooled synapsin and synaptophysin axons are indicated by *yellow dots.* The synapse density in KD neurons was decreased by 15±4% as compared to SCR (p<0.05, n = 76 and 72 SCR and KD axons from 6 independent experiments).

To test whether VGLUT1 expression regulates accumulation of synaptic vesicle proteins at individual presynaptic terminals, transfected axons were identified. Then synaptophysin and synapsin expression were measured at individual presynaptic terminals formed by transfected neurons. Knockdown of VGLUT1 expression resulted in a significant decrease in synaptophysin accumulation at presynaptic puncta (**Figure 7A and B**; p<0.0001; n = 1305 and 978 puncta from 36 and 35 axons for SCR and KD, respectively). A similar decrease in synapsin intensity was also observed in VGLUT1 shRNA-transfected axons when compared to control axons (**Figure 7C and D**; p<0.0001; n = 1802 and 1539 puncta from 57 and 51 axons for SCR and KD, respectively). To further control for off-target effects, control neurons were compared to neurons that expressed both VGLUT1-shRNA and VGLUT1. When VGLUT1 expression levels were equivalent (p = 0.224, based on VGLUT1 immunocytochemistry), synaptophysin expression levels were unchanged (**Figure 7E**; p = 0.940; n = 13 and 9 axons for SCR and KD+VGLUT1, respectively).

Interestingly, the density of presynaptic puncta was also decreased by knockdown of VGLUT1 (**Figure 7F**), with a 15+/−4% decrease in synapse density in knockdown axons (p = 0.012; n = 76 SCR and 72 KD axons). This decrease in synapse density could be seen when presynaptic puncta were identified by either synaptophysin or synapsin immunofluorescence (**Figure 7F**). This decrease in synapse density is unlikely to be due to changes in axon or dendrite elongation or branching since images were collected in regions of axon that would have already grown and their postsynaptic partners were not transfected, although local changes in dendrite growth due to altered glutamatergic transmission could play a role.

## Discussion

Our understanding of the mechanisms that control presynaptic terminal development are only beginning to take form. Here, we tested the hypothesis that VGLUT1 regulates presynaptic terminal development. To do so, we used fluorescence confocal microscopy to examine the dynamics and function of VGLUT1 expression during excitatory synapse development in postnatal cerebral cortical neurons. As synapse development proceeded, a higher percentage of terminals expressed VGLUT1, and VGLUT1 expression increased dramatically at individual terminals. In addition, VGLUT1-positive presynaptic terminals correlated with greater expression of synaptic proteins. Using shRNA targeted to VGLUT1, we disrupted this developmentally regulated increase in VGLUT1 at presynaptic puncta and evaluated the expression of synaptic vesicle proteins. By restricting the VGLUT1 knockdown to a small population of neocortical neurons, we were able to evaluate the cell-autonomous effects of changes in VGLUT1 expression. When VGLUT1 expression was limited, we observed a concomitant reduction in the accumulation of the synaptic vesicle proteins synaptophysin and synapsin at individual terminals, along with a decrease in synapse density. The changes in synaptic protein accumulation observed here could be due to changes in recruitment of synaptic vesicle proteins to presynaptic terminals, changes in synthesis or stability of synaptic proteins, or in the formation, accumulation or retention of synaptic vesicles at terminals. It will be important to explore these potential mechanisms in future studies.

It is well-established that the overall expression of VGLUT1 increases during development, while VGLUT2 decreases [11], [16], [17], [20]. Previous biochemical, immunohistological and electrophysiological studies have reported that VGLUT1 and VGLUT2 can be expressed in the same presynaptic terminals [11], [15], [17], [18], [19]. Interestingly, immunoisolation using VGLUT1 or VGLUT2 antibodies indicated that VGLUT1 and VGLUT2 can even be co-expressed in a subset of synaptic vesicles [19]. Using immunocytochemistry in slices of visual and somatosensory cortex, three studies showed that VGLUT1 and VGLUT2 can be found together in a subset of presynaptic terminals in intact developing postnatal cortex [11], [18], [20], consistent with our observations in cultured visual cortical neurons. Here, we showed that both proteins were expressed in ∼25% of presynaptic terminals, throughout postnatal development of cortical neurons. As synapse density increased, both the fraction of presynaptic terminals that expressed VGLUT1 and the expression of VGLUT1 at terminals increased. In contrast, VGLUT2 expression was only modestly decreased, and the fraction of terminals expressing VGLUT2 did not change. Interestingly, at puncta that expressed both isoforms, the ratio of VGLUT1 to VGLUT2 increased significantly during the period of intense synapse formation. Therefore, we conclude that, during the first 2 weeks postnatal, the developmental switch from VGLUT2 to VGLUT1 occurs primarily through a combination of an increased number of VGLUT1-expressing terminals and increased VGLUT1 at individual terminals.

Together, our data suggest that VGLUT1 regulates presynaptic terminal development in cortical neurons in a cell-autonomous manner. VGLUT1 knockdown in individual neurons resulted in both reduced SV protein accumulation at nascent presynaptic terminals and decreased density of presynaptic terminals made by knockdown neurons. In addition, our data support a direct role of VGLUT1 in presynaptic protein accumulation since network activity was not disrupted. Interestingly, a recent study reported that in the hippocampus of more mature brains (3 weeks postnatal), after the majority of synapses have already formed, overall expression of synapsin and synaptophysin are decreased in VGLUT1 knockout animals [16], implying that the changes we observed during synapse development may persist at mature synapses. However, another study did not find changes in expression of synaptophysin in synaptosomes prepared from VGLUT1 knockout brains at P17 (Wojcik et al., 2004), suggesting that additional studies will be necessary to fully-understand the changes occurring when VGLUT1 is completely absent from neurons.

VGLUT1-dependent regulation of presynaptic development could be due to either (i) altered loading of synaptic vesicles with glutamate resulting in changes in glutamate release or (ii) structural or morphological changes that depend on VGLUT1 expression but are independent of VGLUT1 activity. There are previous reports that are consistent with the observed effects being mediated by glutamate. For example, the level of expression of VGLUT1 can regulate quantal size and, therefore, the level of glutamate receptor activation [15], [25], [26]. Furthermore, blockade of glutamate receptor activation induces a reduction in presynaptic protein accumulation at nascent presynaptic terminals [27] that is similar to that observed here. Although we are unaware of any studies demonstrating a function of VGLUT1 that is independent of its loading activity, such structural roles have been reported for other synaptic transmembrane proteins, such as the role of NMDA receptors in spine stability [36] or AMPA receptors in presynaptic terminal stability [37]. It will be important to distinguish between these potential mechanisms in the future.

It is well-established that activity plays an essential role in the establishment of cortical circuitry, and activity bi-directionally controls VGLUT1 expression [17], [27]. Regulation of VGLUT1 expression or function might be utilized by developing neurons as a mechanism for activity-dependent synapse development during circuit formation. In addition to its relevance to normal development of cortical circuits, the relationship between VGLUT1 expression and presynaptic development might be relevant for diseases such as anxiety and depression. Anxiety and depression have been linked to synapse formation and circuit development [38], [39], [40], [41], [42], [43], and a series of recent clinical and animal studies have also linked glutamatergic signaling and depression [44]. Interestingly, chronic stress decreases VGLUT1 [45], and several anti-depressants and anxiolytics increase VGLUT1 expression in the cortex [46], [47]. Regulation of synapse development via VGLUT1 might provide a novel target for new therapeutics for anxiety and depression.
